# An exciting time for genome editing: an interview with David R. Liu

**DOI:** 10.1093/nsr/nwy146

**Published:** 2018-11-23

**Authors:** Weijie Zhao, Kevin T Zhao

**Affiliations:** Weijie Zhao is an NSR news editor based in Beijing; Kevin T. Zhao is a graduate student in David Liu's group at Harvard University and the Broad Institute

## Abstract

In 1987, several Osaka University researchers discovered a special kind of clustered DNA repeats in bacteria. Within a few years, two other groups independently discovered the same phenomenon but no one knew its function at the time. Only a small handful of scientists studied this property from its discovery in 1987 to 2005. It was then that the function of these DNA repeats, which were named Clustered Regularly Interspaced Short Palindromic Repeats (CRISPR), was finally elucidated. Researchers found that CRISPR, when combined with its CRISPR-associated partner (Cas), is crucial for the functioning of the bacterial adaptive immune system against viral phage infection. CRISPR sequences can be transcribed into targeting RNA molecules, and Cas enzymes are guided by these RNAs to cut specific viral DNA loci, rendering resistance against the viral infection.

Scientists realized that this natural bacterial immune response system could be engineered to become a powerful genome editing tool. Prior to CRISPR, existing genome editing tools such as Zinc Finger Nucleases (ZFNs) and Transcription Activator-Like Effector Nucleases (TALENs) relied solely upon protein–DNA interactions to target an enzyme to specific DNA sequences. The design, engineering and evolution of proteins for various DNA sequences is difficult and time-consuming. In contrast, the CRISPR-Cas system uses Watson–Crick base pairing between a guide RNA and the target DNA to localize the complex to specific DNA sequences. This feature enables users to simply change an RNA sequence to match a DNA target to reposition the whole complex.

Since then, numerous talented scientists have headed into this field. Within a single decade, they have developed the CRISPR-Cas system into a powerful genome editing tool and applied it to the editing of microorganisms, plants, animals and even human embryos. David R. Liu, Professor of Harvard University and the Broad Institute, and an investigator of the Howard Hughes Medical Institute, is one of them. One of his major contributions to the field is the development of ‘base editing’. His group engineered the CRISPR system to transform it from being DNA scissors that cut DNA into specific DNA base pair rewriters that directly convert one base pair to a different base pair. This development opens the door to precision genome editing, raising the possibility of treating thousands of genetic diseases that are caused by single point mutations in the human genome. Here, David talks about this exciting time for genome editing.

**NSR:** What have been the major developments of the CRISPR system in recent years?

**Liu:** There have been many seminal developments in CRISPR biology and in the application of CRISPR systems. Many of those developments involved the elucidation of the natural mechanisms and components of this bacterial immune system. Those biological discoveries have enabled a renaissance in genome editing based on the use of CRISPR to manipulate genomes and epigenomes in many ways, including cutting DNA, base editing, turning on genes, turning off genes and more.

There will certainly be other significant developments in the future. Many talented and dedicated researchers are working on the biology of CRISPR components and on developing novel genome editing systems. For the foreseeable future, these systems will continue to be improved, and novel systems will continue to be discovered that will bring new capabilities to the life sciences. That is the reason why it is such an exciting time to be involved in this kind of research.

**Figure 1:**
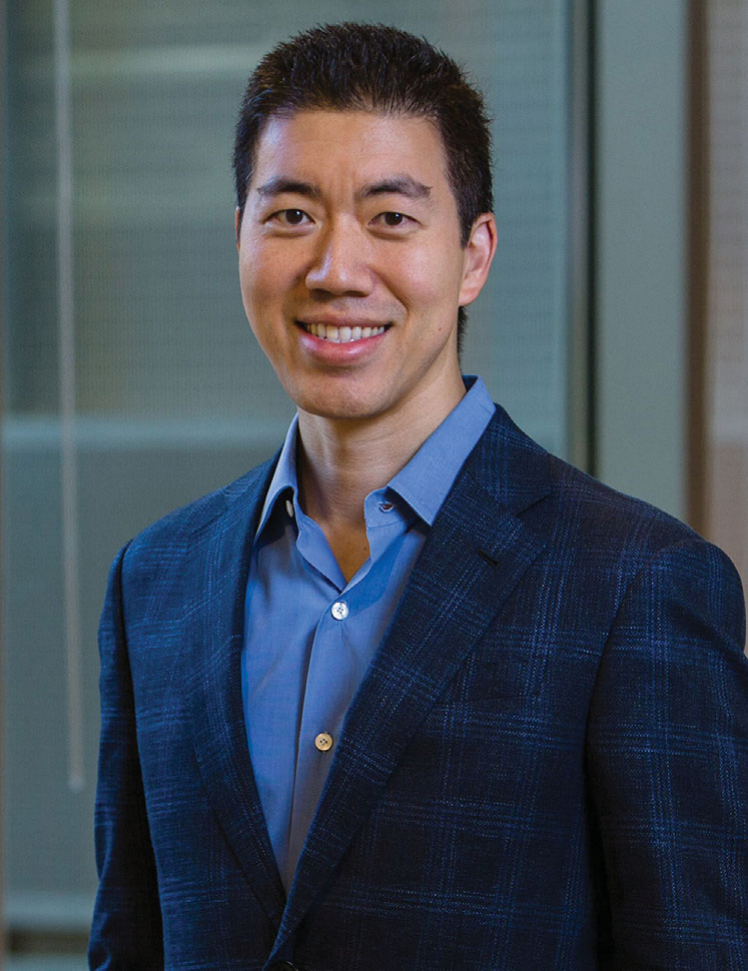
Professor David R. Liu is one of the pioneering scientists in the field of genome editing. *(Courtesy of David R. Liu)*

**NSR:** What are the challenges facing CRISPR?

**Liu:** In some cases, it can be difficult to target CRISPR to exactly the right position of the genome. In other cases, we worry about the difficulty of delivering the CRISPR agents to the right cells, especially in a patient or in an animal. We also must understand off-target editing and we need to characterize the consequences of these off-target editing events. Before CRISPR is used to treat patients with genetic diseases, we have to ensure that it is safe and effective.

No single tool will be able to address all needs for all applications.

—David R. Liu

**NSR:** What about the other genome editing tools? Will they be replaced by CRISPR?

**Liu:** Genome editing is a very broad field. When we apply genome editing to different fields, for example in agriculture or in medicine, we need different kinds of tools to deal with different kinds of problems. No single tool will be able to address all needs for all applications. For example, if your goal is to make a targeted point mutation of a certain type, then base editors can offer many advantages. But if your goal is to insert a big chunk of DNA or to cut out a big chunk of DNA, then base editors are not a useful tool for doing those things. If you are editing human patients, you need to ensure that undesired edits are infrequent enough that they are unlikely to do harm to the patient. But if you are editing plants to generate improved crops, then undesired edits might be easily avoided by simply screening 100 different seedlings. Therefore, we will always need a broad collection of tools to enable the broadest possible benefits to society. I anticipate that ZFNs, TALENs, CRISPR-Cas9 nucleases, base editors, epigenome-modifying agents and additional forms of genome editing tools will each have an important role in allowing the field to reach its aspirations.

**NSR:** Will new and more powerful genome editing tools appear in the future?

**Liu:** Yes. History tells us that every year in the genome editing field, there have been new tools discovered that continue to define the state-of-the-art in the field. So it is easy to predict that new genome editing tools will continue to increase our capabilities, and in some cases to improve upon older tools.

**NSR:** How about the current progress of applying these tools in a clinical setting?

**Liu:** As you probably know, CRISPR has already entered human clinical trials in some countries. Both *ex vivo* and *in vivo* genome editing for human therapies are quite promising based on recent animal studies. These tools could represent a new generation of human medicine that treats diseases with a genetic component, not by just treating the symptoms, but by actually treating the root cause of the disease. It is a very exciting possibility, but one that must be developed very carefully, with the full engagement of scientists, ethicists, and regulatory communities around the world.

**NSR:** When will genome editing therapies be approved for common patients?

**Liu:** I would be surprised and disappointed if there were not extensive clinical trials within the next 5 years, and hopefully some approved drugs within the next 5–10 years. The diseases that will likely be targeted first include grievous ones that inflict a great deal of suffering or loss of life, with no known effective treatment. The next decade will be a very exciting one to observe how these breakthroughs in CRISPR biology and genome editing tool development translate into better outcomes for patients.

**NSR:** There are many misinterpretations of genome editing among the public. How could scientists better communicate with the public?

**Liu:** That is a very important point. Scientists need to help the public understand genome editing and the capabilities of CRISPR systems. Equally important, scientists should do a better job educating the public about basic human genetics. Many of the questions we get asked, like ‘can you use CRISPR to create a hyperintelligent baby or a very athletic human?’, reflect a lack of understanding of human genetics.

There is no single gene that defines your intelligence. In fact, a trait as complicated as intelligence is probably the result of thousands of genes as well as environmental conditions. So in my opinion, genome editing is unlikely to make a designer baby that has perfect intelligence or the like. Also, the effort, expense and resources needed to develop and test genome editing in people is enormous. If you have a child with a genetic disease and the prognosis is that the child will die in a short period of time, that is a very serious problem. People will justifiably invest enormous resources to treat that kind of afflicted population, but to make somebody's hair shinier, make them run faster or perform music better? I doubt such changes will ever be a goal of genome editing researchers.

**NSR:** Some researchers have tried to make animals with more muscles.

**Liu:** Yes. But that has already been done for many years by breeding, without any need for genome editing. Natural mutations in *MSTN* (the gene encodes myostatin) and other genes naturally lead to animals that have more muscles. Genome editing just provides us with a faster way to install some of the changes that nature has already revealed.

Before genome editing, we had already practiced selective breeding, which is a primitive but effective way to manipulate the genome of animals and plants, for thousands of years. I hope that the public appreciates that the purposeful manipulation of genes is not new to genome editing. Humans have been manipulating genomes through selective breeding for thousands of years. Genome editing simply provides us with more efficient and precise ways to modify genomes.

**NSR:** How can these messages get transferred to the public?

**Liu:** Certainly, scientists can and should take opportunities to directly engage the public. But I think we also need the government to organize mechanisms to make sure that the most important information around these major developments is in the minds of the public. Not only do scientists have a responsibility, but the entire citizenry, including the government, has a responsibility to make informed and thoughtful decisions about these important issues.

[Clinical trails] must be developed very carefully, with the full engagement of scientists, ethicists, and regulatory communities around the world.

—David R. Liu

There is a natural and very productive partnership between genome editing and enzyme evolution.

—David R. Liu

**NSR:** What are your current research interests?

**Liu:** Our group integrates chemistry and evolution to study biology, and to enable new kinds of medicines. We actively research three areas. Our first area has developed and uses DNA-templated synthesis to discover synthetic small molecules and synthetic polymers that have interesting biological properties. Our second area has developed and uses protein evolution, including phage-assisted continuous evolution, or PACE, to evolve proteins that have properties of interest to researchers. And of course, base editing and genome editing is our third area. All of these research areas seek to enable new treatments for patients with variety of diseases, including some genetic diseases.

**NSR:** The 2018 Nobel Prize was awarded to enzyme evolution. Is it related to your research, and how can it facilitate genome editing?

**Liu:** Our group integrates genome editing and protein evolution. We have used protein evolution to evolve new variants of TALEN proteins, CRISPR-Cas9 proteins and base editors that have new useful properties.

There is a natural and very productive partnership between genome editing and enzyme evolution. The genome editing proteins that occur in nature are not evolved to have the properties optimal for use by scientists and doctors. So in order to give naturally occurring proteins special properties that make them more useful for genome editing, we use protein engineering and protein evolution to generate more useful forms of these proteins.

**NSR:** Everybody believes that CRISPR will win a Nobel Prize in several years. So who are the significant people in this field and the possible candidates?

**Liu:** There are too many people who have made major contributions to this field and it is not useful to try to name them all in a short interview.

But I certainly agree with the premise behind the question, in that because genome editing has had such a transformative impact on science and is starting to have an impact on society, it is deserving of a Nobel Prize one day. But I also hope that people appreciate that having prizes awarded or not awarded to certain scientific discoveries does not always mirror the importance of those discoveries.

